# Supplementary figures and images for: Novel use of XSTAT 30 for mitigation of lethal non-compressible torso hemorrhage in swine
Source: PLoS One. 2020 Nov 18;15(11):e0241906. doi: 10.1371/journal.pone.0241906 (PMC7673511; doi:10.1371/journal.pone.0241906)

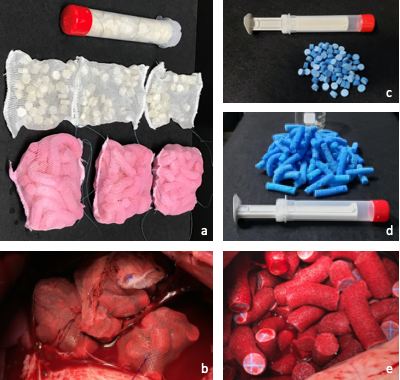

Supplement: S1 Fig — Subpanel a: Compact and expanded pellets in radiopaque bags. Subpanel b: Pellets in radiopaque bags in-situ. Subpanel c: Compact free pellets. Subpanel d: Expanded free pellets. Subpanel e: Expanded free pellets in-situ. (TIFF) [file pone.0241906.s001.tiff]
